# Body Mass Index Status across Different Psychiatric Disorders in a National Survey amongst Children and Adolescents: To Identify the Role of Gender

**Published:** 2019-10

**Authors:** Mohammad Reza Mohammadi, Seyed-Ali Mostafavi, Zahra Hooshyari, Ali Khaleghi, Nastaran Ahmadi

**Affiliations:** 1 Psychiatry and Psychology Research Center, Tehran University of Medical Sciences, Tehran, Iran.; 2 Yazd Cardiovascular Research Center, Shahid Sadoughi University of Medical Sciences, Yazd, Iran.

**Keywords:** *Adolescent*, *Body Mass Index*, *Child*, *Gender*, *Psychiatric Disorders*

## Abstract

**Objective:** Body mass index undergoes a substantial change in some psychiatric disorders. This study aimed to explore the status of body mass index (BMI) in different psychiatric disorders in a national survey among children and adolescents and to identify the role of gender in this regard.

**Method** **:** A total of 30 532 children and adolescents were randomly selected using cluster sampling method with equal blocks of three age groups and two genders. Psychiatric disorders were assessed using a standardized face-to-face diagnostic interview of Kiddie Schedule for Affective Disorders and Schizophrenia-Present and Lifetime Version (KSADS-PL), and Body Mass Index (BMI) was measured for each participant.

**Results: **In this study, 22 730 children and adolescents (109 46 boys and 11784 girls), with valid data of BMI, completed the KSADS-PL interview. The prevalence of psychiatric disorders was 21.2% among underweight participants, 22.8% among overweight participants, and 22.2% among obese participants, which was significantly higher than normal weight participants with 19.6% (X2 = 17.55; p = 0.001). In boys’ subgroup, depression and separation anxiety were mostly seen among the underweight category, while tic disorder was mostly seen in the obese category. In girls’ subgroup, on the other hand, generalized anxiety was mostly observed in the underweight category, while oppositional defiant disorder (ODD), depression, and mental retardation were mostly observed in the obese category. In total, the highest mean BMI rates were among the children and adolescents with alcohol abuse disorder, mania, and panic disorder. However, the lowest BMI rates were among those with attention deficit hyperactivity disorder (ADHD), separation anxiety disorder (SAD), and enuresis.

**Conclusion: **This study gives an overall picture of BMI status in different psychiatric disorders according to gender. Furthermore, in a multidisciplinary approach, the results of this study drew the attention of child psychiatrists to the status of BMI in their clients.

Psychiatric disorders and body mass index (BMI) problems are both major global health concerns (1). These two groups of disorders are important causes of preventable mortality and morbidity. Psychiatric disorders contribute to 7% of the global burden of disease as Disability-Adjusted Life Years (DALY) and 19% to all years lived with disability (2). In Iran, depression is among the 10 leading causes of DALYs (3). On the other hand, obesity and overweight were among the most important causes of years of life lost (YLL) in Iran (4). 

In all, these two clusters of disorders increase the mortality and morbidity rates spatially when they occur together (2, 5). Furthermore, they impose a considerable burden to the patient, their family, and the health care system (5). Also, they increase the risk of development of cardiovascular diseases, diabetes, and some types of cancers in later life (6, 7). Additionally, the disability and costs are considerably increased when the disorders start in early life and rename without treatment (8-11). 

Psychiatric disorders and BMI disturbances have great rates of comorbidities (12).

Previous studies have tried to clarify the association between BMI and some psychiatric disorders but the results bring about conflicting answers and raise new questions. For example, some studies have reported increased BMI (13) and some others have reported decreased BMI as a result of mood disorders (14). Another study has revealed that adolescents with depression have 70% higher risk of developing obesity (15). Recent studies have focused on the role of gender in different patterns of association of mood disorders and BMI (16). In another study on the association between psychiatric disorders and obesity, investigators revealed 27% increased risk of panic disorder among obese individuals (12). Darby et al reported an increasing trend in the comorbidity of eating disorders and obesity (17). McCarty et al reported an increased risk of obesity only among female alcohol users (18). However, conflicting results exist regarding attention deficit hyperactivity disorder (ADHD). Cortese et al reported 44% increase in the risk of obesity as a result of adult persistent ADHD. Considering the confounding factors, a positive association was observed only among women (9% increased risk of obesity), but the association was negative in men (19). The conflicting results in different studies may be due to differences in the study sample, methodologies, and lack of control over confounders. 

Gender differences are suspected to be the most important moderator in the association between most of psychiatric disorders and BMI status (20). Nevertheless, in most previous studies on the association between psychiatric disorders in children and adolescents and BMI, the cofactor role of sex has been ignored. Most previous studies have focused on common psychiatric disorders such as depression and anxiety disorders, while the association of other psychiatric disorders and BMI is usually overlooked. Moreover, previous studies have assessed psychiatric disorders per se, which might have been misdiagnosed by using a simple questionnaire rather than face-to-face structured interview.

Understanding the status of BMI in patients with different psychiatric disorders is clinically important for managing the disease and its complications. However, little is known about BMI changes in different psychiatric disorders. Hence, due to the lack of knowledge in this field, the present study aimed to investigate the status of body mass index among children and adolescents with different psychiatric disorders in a large scale survey and to study the role of gender in this regard.

## Materials and Methods

***Data Set***

The data set for the present study was taken from that of Iranian Children and Adolescents' Psychiatric Disorders Survey from September 22, 2016 through January 3, 2018 (21), which was performed in all provinces of Iran and have screened 30 532 children and adolescents aged 6-18 years for psychiatric disorders. 

***Sampling***

The samples were selected using cluster random sampling method. In each province, 170 clusters were randomly selected according to postal code (A total of 340 clusters were randomly selected in Tehran.). Each cluster was blocked by equal number of boys and girls and equal samples in age range of 6-10, 10-14, and 14-18 years. Also, clusters were selected from urban and rural areas proportional to size of children and adolescents living in the urban and rural areas in each province based on the last national census. Then, 1020 participants were randomly selected from each province (2040 participants in Tehran). The inclusion criteria were Iranian nationality and age range of 6 to 18 years. The exclusion criterion was history of comorbid severe physical disorders.

***Study Design and Procedure***

In this large scale cross sectional survey, the total of 250 trained clinical psychologists referred to the houses randomly selected according to postal codes in all provinces. The movements of interviewers according to clusters were monitored with GPS from the project management’s central office. The interviewers first introduced themselves and described the study’s procedure and objectives. Then, they obtained written informed consent forms. Afterward, the clinical psychologists performed face-to-face interviews according to the Diagnostic and Statistical Manual of Psychiatric Disorders Criteria- Fourth Edition (DSM-IV). Furthermore, the accuracy of the interviews and the diagnosis were randomly checked by the project management’s central office by phone. 

***Diagnosis of Psychiatric Disorders***

The semi-structured interview of Kiddie Schedule for Affective Disorders and Schizophrenia-Present and Lifetime Version (K-SADS-PL) was used to diagnose common psychiatric disorders in children and adolescents, based on DSM-IV. Also, the Persian version of KSADS-PL has been validated in the population under study. The test-retest reliabilities of different categories of this tool were between 0.56 to 0.81(22). The Kappa for interrater reliability of all K-SADS-PL categories was between 0.66 to 0.81 (p < 0.009), which indicates a good interrater reliability and agreement between interviewers. This tool is able to diagnose depressive disorders, mania, hypomania, psychosis, panic disorder, separation anxiety disorder (SAD), social phobia, specific phobias, agoraphobia, generalized anxiety disorder (GAD), obsessive compulsive disorder (OCD), posttraumatic stress disorder (PTSD), attention deficit hyperactivity disorder (ADHD), oppositional defiant disorder (ODD), conduct disorder (CD), tic disorder (TD), autism, mental retardation (MR), epilepsy, alcohol abuse, and enuresis. The sensitivity of the Persian version of K-SADS-PL for diagnosing different psychiatric disorders was between 78.9 to 100 and the specifity was between 79.6 to 100 (22). The clinical psychologists were trained in the central office of each province. The diagnostic screening interview for each child lasted for 45 to 120 minutes. 

***BMI Measurements and Classifications***

Standard BMI was measured using the Quetelet index, dividing the weight of individuals in kilograms by their height in meters squared. In this study, the national cutoff points were used to categorize BMI, and obesity was defined as ≥ 95th percentile of the national age and sex specified cutoffs for BMI. Also, overweight was defined as < 95th to ≥ 85th percentile of national age and sex specified cutoffs for BMI; normal weight was defined as < 85th to ≥ 5th percentile of national age and sex specified cutoffs for BMI; and defined underweight was set at < 5th percentile of national age and sex specified cutoffs for BMI (23).

***Statistics***

The data were analyzed using SPSS version 19 (IBM Corp. Released 2010. IBM SPSS Statistics for Windows, Version 19.0. Armonk, NY: IBM Corp). The comparison of prevalence rates of different psychiatric disorders in children and adolescents across the BMI categories is done by chi-squared test separately in two genders. Furthermore, t test was used to compare the mean BMI of patients in each psychiatric disorder with mean BMI in healthy children and adolescents. P value less than 0.05 was considered significant.

***Ethics***

The ethical principles of declaration of Helsinki in 1964 and its later revisions were met in this study. After describing the study procedure and objectives, the interviewers obtained written informed consent from parents and adolescents. Participants were agreed to participate and their parents consented on their behalf. They were assured that the research team would keep their information confidential. Furthermore, the ethics committee review board in the National Institute for Medical Research and Development (NIMAD) approved the study protocol (ethics code: IR.NIMAD.REC.1395.001).

## Results

In total, the valid data of 22 730 participants were analyzed (10 946 boy and 11 784 girls). Table 1 shows the prevalence of psychiatric disorders among children and adolescents according to BMI category.

Depression and separation anxiety were mostly seen among underweight boys, while tic disorder was mostly observed in obese boys and enuresis in overweight boys. Furthermore, the boys with at least one psychiatric disorder were mostly overweight (Table 2). 

On the other hand, generalized anxiety was mostly observed in underweight girls, while ODD, depression, and mental retardation were mostly observed in obese girls. Similar to boys, girls with at least one psychiatric disorder were overweight (Table 3). Furthermore, the table 4 shows the comparison of mean BMI in each psychiatric disorder with mean BMI in children and adolescents without any psychiatric disorders.

**Figure 1:**
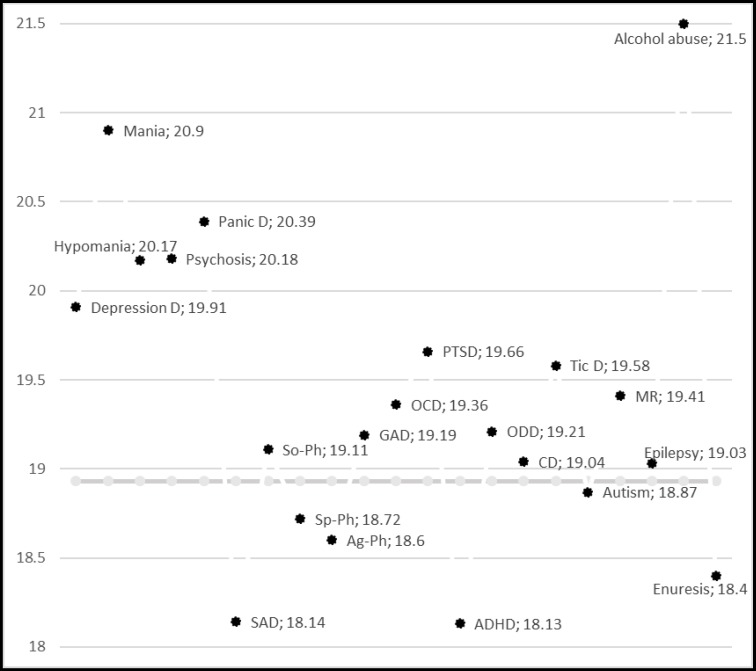
Plan of Mean BMI in Psychiatric Disorders Compared to Each Other and Normal BMI Line

**Figure 2:**
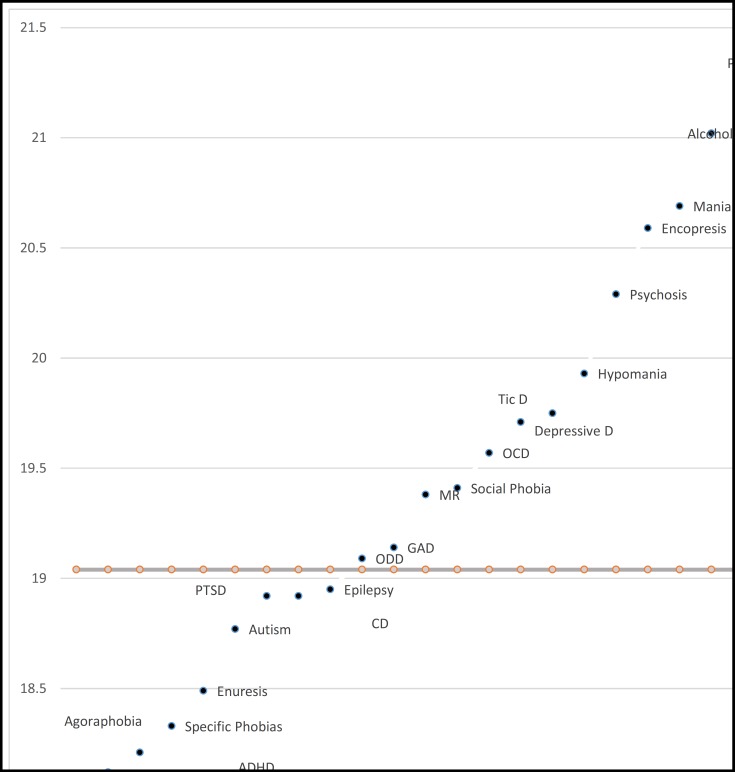
Plan of Mean BMI in Psychiatric Disorders in Boys Compared to Each Other and Normal BMI line

**Figure 3:**
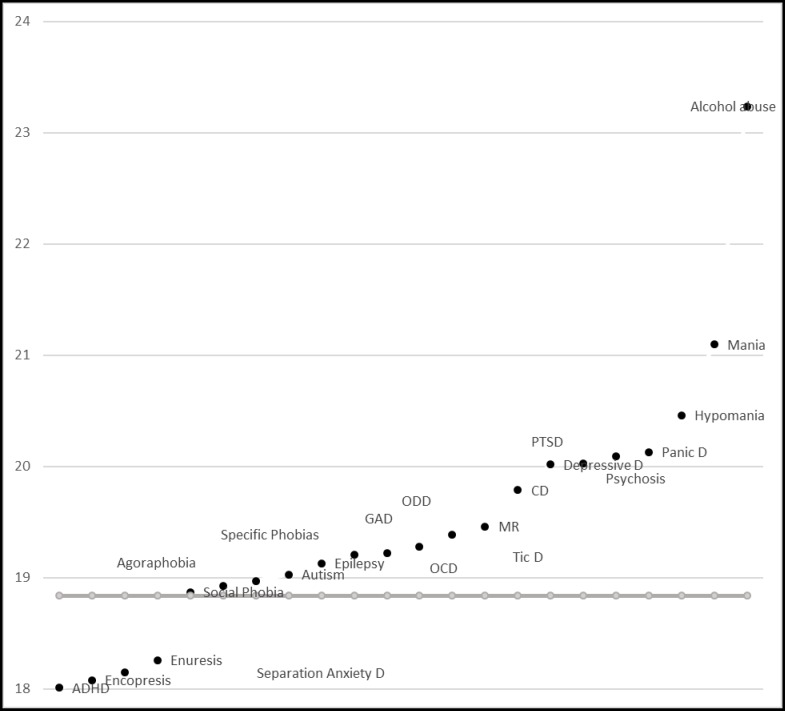
Plan of Mean BMI in Psychiatric Disorders in Girls Compared to Each Other and Normal BMI line

Figure 1 shows that the highest BMI rates were observed among children and adolescents with alcohol abuse disorder, mania, and panic disorder. However, the lowest BMI rates were found among those with ADHD, SAD, and Enuresis. Figure 2 and 3 show the pattern of mean BMI in each psychiatric disorder in comparison with each other and normal BMI line.

**Table 1:** Prevalence of Psychiatric Disorders among Children and Adolescents According to BMI

	**BMI Categorized Based on the National Cutoff Points**					**X** ^2^	**P value**
	**Underweight**	**Healthy weight**	**Overweight**	**Obese**	**Total**		
	**N(P)**	**N(P)**	**N(P)**	**N(P)**	**N(P)**		
Depressive Disorders	34(3.1)	343(2)	39(1.8)	29(2.9)	445(2.1)	9.903	0.019*
Mania		16(0.1)	2(0.1)	2(0.1)	20(0.1)	2.223	0.527
Hypomania	1(0.1)	34(0.2)	4(0.2)	2(0.2)	41(0.2)	0.621	0.892
Psychosis	3(0.3)	45(0.3)	2(0.1)	5(0.5)	55(0.2)	4.529	0.210
Panic Disorder	1(0.1)	30(0.2)	3(0.1)	3(0.3)	37(0.2)	1.491	0.684
Separation Anxiety	60(5.3)	800(4.5)	117(5.3)	54(5.2)	1031(4.6)	4.937	0.176
Social Phobia	20(1.7)	344(1.9)	44(2)	20(1.9)	428(1.9)	0.220	0.974
Specific Phobias	42(3.7)	540(3)	79(3.6)	29(2.8)	690(3.1)	3.678	0.298
Agoraphobia	28(2.5)	359(2)	52(2.3)	19(1.8)	458(2.1)	2.355	0.502
Generalized Anxiety	39(3.4)	449(2.5)	66(3.0)	22(2.1)	576(2.6)	5.918	0.116
Obsessive Compulsive Disorder	33(2.9)	462(2.6)	65(2.9)	26(2.5)	586(2.6)	1.372	0.712
Posttraumatic Stress Disorder	7(0.6)	118(0.7)	17(0.8)	7(0.7)	149(0.7)	0.403	0.940
Attention Deficit Hyperactivity Disorder	51(4.5)	621(3.5)	87(4)	46(4.4)	805(3.6)	6.110	0.106
Oppositional Defiant Disorder	44(3.9)	654(3.7)	101(4.6)	47(4.5)	846(3.8)	5.991	0.112
Conduct Disorder	10(0.9)	144(0.8)	16(0.7)	9(0.9)	179(0.8)	0.277	0.964
Tic Disorder	16(1.4)	217(1.2)	42(1.9)	20(1.9)	295(1.3)	10.097	0.018*
Autism	2(0.2)	15(0.1)	6(0.3)	1(0.1)	24(0.1)	6.918	0.075
Mental retardation	18(1.5)	190(.1)	37(1.6)	21(2)	266(1.2)	14.238	0.003*
Epilepsy	32(2.7)	421(2.3)	53(2.3)	25(2.4)	531(2.3)	1.510	0.680
Alcohol abuse		24(0.1)	3(0.1)	1(0.1)	28(0.1)	1.614	0.656
Enuresis	48(4.2)	795(4.4)	135(6.1)	47(4.5)	1025(4.6)	12.615	0.006*
At least one psychiatric disorders*	248(21.2)	3568(19.6)	517(22.8)	235(22.2)	4568(20.1)	17.552	0.001*
Total	1172(5.2)	18239(80.2)	2264(10)	1058(4.7)	22730(100)		

BMI: Body Mass Index

**Table 2:** Prevalence of Psychiatric Disorders among Boys According to BMI

	**BMI Categorized Based on the National Cutoff Points**					**X** ^2^	**P value**
	**Underweight**	**Healthy ** **weight**	**Overweight**	**Obese**	**Total**		
	**N(P)**	**N(P)**	**N(P)**	**N(P)**	**N(P)**		
Depressive Disorders	18(3.6)	136(1.7)	17(1.6)	13(2.6)	184(1.8)	12.534	0.006*
Mania	-	8(0.1)	1(0.1)	1(0.1)	10(0.1)	1.079	0.782
Hypomania	1(0.2)	19(0.2)	2(0.2)	1(0.2)	23(0.2)	0.108	0.991
Psychosis	3(0.6)	23(0.3)	1(0.1)	4(0.8)	31(0.3)	7.564	0.056
Panic Disorder	-	6(0.1)	1(0.1)	1(0.2)	8(0.1)	1.488	0.685
Separation Anxiety	32(6.1)	355(4.2)	64(5.8)	23(4.6)	474(4.4)	9.655	0.022*
Social Phobia	8(1.5)	158(1.8)	19(1.7)	9(1.8)	194(1.8)	0.374	0.946
Specific Phobias	18(3.4)	211(2.5)	30(2.7)	12(2.4)	271(2.5)	2.006	0.571
Agoraphobia	13(2.5)	144(1.7)	21(1.9)	6(1.2)	184(1.7)	2.845	0.416
Generalized Anxiety	15(2.9)	187(2.2)	26(2.3)	13(2.6)	241(2.3)	1.266	0.737
Obsessive Compulsive Disorder	15(2.8)	193(2.3)	29(2.6)	13(2.6)	220(2.3)	1.295	0.730
Posttraumatic Stress Disorder	3(0.6)	45(0.5)	4(0.4)	3(0.6)	55(0.5)	0.635	0.888
Attention Deficit Hyperactivity Disorder	28(5.4)	390(4.6)	60(5.4)	27(5.4)	505(4.7)	2.570	0.463
Oppositional Defiant Disorder	25(4.8)	379(4.4)	56(5)	23(4.6)	483(4.5)	0.952	0.813
Conduct Disorder	10(1.9)	124(1.5)	13(1.2)	8(1.6)	155(1.5)	1.362	0.714
Tic Disorder	8(1.5)	141(1.6)	27(2.4)	17(3.4)	193(1.8)	10.759	0.013*
Autism	1(0.2)	10(0.1)	3(0.3)	1(0.2)	15(0.1)	1.859	0.602
Mental retardation	8(1.5)	102(1.2)	20(1.8)	8(1.6)	138(1.3)	3.456	0.326
Epilepsy	13(2.4)	181(2.1)	17(1.5)	10(1.9)	221(2)	2.127	0.546
Alcohol abuse		20(0.2)	1(0.1)	1(0.2)	22(0.2)	2.141	0.544
Enuresis	31(5.9)	484(5.6)	89(8)	30(5.9)	634(5.9)	9.583	0.022*
At least one psychiatric disorders*	123(22.8)	1829(20.9)	274(24.1)	120(23.3)	2346(21.4)	7.852	0.049*
Total	540(4.9)	8753(80)	1139(10.4)	514(4.7)	10946		

BMI: Body Mass Index

**Table 3:** Prevalence of Psychiatric Disorders among Girls According to BMI

	**BMI Categorized Based on the National Cutoff Points**					**X** ^2^	**P value**
	**Underweight**	**Healthy weight**	**Over weight**	**Obese**	**Total**		
	**N(P)**	**N(P)**	**N(P)**	**N(P)**	**N(P)**		
Depressive Disorders	16(2.7)	207(2.3)	22(2.1)	15(2.9)	260(2.3)	1.256	0.740
Mania		8(0.1)	1(0.1)	1(0.2)	10(0.1)	1.146	0.766
Hypomania		15(0.2)	2(0.2)	1(0.2)	18(0.2)	1.058	0.787
Psychosis		22(0.2)	1(0.1)	1(0.2)	24(0.2)	2.364	0.500
Panic Disorder	1(0.2)	24(0.3)	2(0.2)	2(0.4)	29(0.2)	0.736	0.865
Separation Anxiety	28(4.6)	445(4.8)	53(4.9)	31(5.8)	557(4.8)	1.266	0.737
Social Phobia	12(1.9)	186(2)	25(2.3)	11(2)	234(2)	0.391	0.942
Specific Phobias	24(3.9)	329(3.5)	49(4.5)	17(3.2)	419(3.6)	2.966	0.397
Agoraphobia	15(2.4)	215(2.3)	31(2.8)	13(2.4)	274(2.4)	1.176	0.759
Generalized Anxiety	24(3.9)	262(2.8)	40(3.7)	9(1.7)	335(2.9)	7.631	0.054*
Obsessive Compulsive Disorder	18(3)	269(2.9)	36(3.3)	13(2.4)	336(2.9)	1.001	0.801
Posttraumatic Stress Disorder	4(0.7)	73(0.8)	13(1.2)	4(0.8)	94(0.8)	2.187	0.534
Attention Deficit Hyperactivity Disorder	23(3.8)	231(2.5)	27(2.5)	19(3.5)	300(2.6)	5.652	0.130
Oppositional Defiant Disorder	19(3.1)	275(3)	48(4.4)	25(4.7)	367(3.2)	10.338	0.016*
Conduct Disorder		20(0.2)	3(0.3)	1(0.2)	24(0.2)	1.554	0.670
Tic Disorder	8(1.3)	76(0.8)	15(1.4)	3(0.6)	102(0.9)	5.313	0.150
Autism	1(0.2)	5(0.1)	4(0.4)		10(0.1)	7.901	0.053*
Mental retardation	10(1.6)	88(0.9)	17(1.5)	13(2.4)	128(1.1)	14.151	0.003*
Epilepsy	6(0.9)	145(1.5)	16(1.4)	10(1.8)	177(1.5)	1.816	0.612
Alcohol abuse		4(0.05)	2(0.2)			4.378	0.223
Enuresis	17(2.8)	311(3.3)	46(4.2)	17(3.2)	391(3.4)	2.982	0.394
At least one psychiatric disorders*	125(19.8)	1739(18.3)	243(21.6)	115(21.1)	2222(18.9)	9.405	0.024*
Total	632(5.4)	9483(80.5)	1125(9.5)	544(4.6)	11784(100)		

BMI: Body Mass Index

**Table 4:** Comparison of Mean BMI in Each Psychiatric Disorder with Mean BMI in Children and Adolescents without any Psychiatric Disorders

	**N**	**µ(std)**	**t test**	**p value**
Depressive Disorders	444	19.89(3.89)	5.463	<0.001**
Mania	20	20.90(3.66)	2.357	0.018*
Hypomania	41	20.17(3.56)	2.119	0.034*
Psychosis	55	20.18(4.11)	2.468	0.014*
Panic Disorder	37	20.39(3.66)	2.375	0.018*
Separation Anxiety	1031	18.14(3.73)	7.014	<0.001**
Social Phobia	428	19.11(3.79)	0.982	0.326
Specific Phobias	690	18.71(3.74)	1.591	0.112
Agoraphobia	458	18.60(3.77)	1.951	0.051
Generalized Anxiety	576	19.19(3.81)	1.685	0.092
Obsessive Compulsive Disorder	586	19.36(3.61)	2.809	0.005*
Posttraumatic Stress Disorder	149	19.66(3.72)	2.391	0.017*
Attention Deficit Hyperactivity Disorder	805	18.13(3.71)	6.232	<0.001**
Oppositional Defiant Disorder	850	19.22(3.91)	2.285	0.022*
Conduct Disorder	179	19.04(3.91)	0.386	0.699
Tic Disorder	295	19.58(3.91)	2.991	0.003*
Autism	24	18.87(4.35)	0.091	0.927
Mental retardation	266	19.41(3.99)	2.114	0.035*
Epilepsy	398	19.03(3.90)	0.523	0.601
Alcohol abuse	28	21.50(2.89)	3.629	<0.001**
Enuresis	1025	18.40(3.86)	4.707	<0.001**
Healthy children and adolescents	18161	18.95(3.69)		
Total	22729	18.93(3.72)		

BMI: Body Mass Index

## Discussion

In this study, the rate of different psychiatric disorders was analyzed according to BMI categories, which showed a significant increase of psychiatric disorders within specific layers of BMI. The results of this study showed that children and adolescents diagnosed with at least one of the psychiatric disorders are significantly more prone to be overweight or obese. Vila et al in a study of mental disorders in obese children and adolescents found that 56.7% of the participants were diagnosed with at least one DSM-IV psychiatric disorders, about 40% experienced anxiety, and 12.2% experienced affective disorder (24). Rankin et al have also revealed multiple psychological comorbidities in children and adolescents with overweight or obesity (25).

Mannan et al reported that adolescents with mood disorders are 70% more prone to be obese (15). De wit et al proposed a u-shaped association between depression and BMI (26) and later point out the gender as the modifier of depression and obesity association (27). In another paper, mohammadi et al reported that boys with depression are usually underweight, while girls with depression are usually obese (16). Even another study claimed that obesity lowers the risk of depression in males but increases the risk of depression in females (18). These results are in line with those of the present study. Here, higher rates of depression was observed among underweight boys and obese girls, which reveals that gender plays an important moderating role in the association between psychiatric disorders, especially depression and BMI. 

BMI: Body Mass Index, Depression D: depression disorder, Panic D: panic disorder, SAD: separation anxiety disorder, So-Ph: social phobia, Sp-Ph: specific phobias, Ag-Ph: agoraphobia, GAD: generalized anxiety disorder, OCD: obsessive compulsive disorder, PTSD: posttraumatic stress disorder, ADHD: attention deficit hyperactivity disorder, ODD: oppositional defiant disorder, CD: conduct disorder, Tic D: tic disorder, MR: mental retardation.

Separation anxiety disorder was mostly seen among underweight boys and generalized anxiety disorder in underweight girls. Unlike our results, Rofey et al in a study with a different method, had followed children with anxiety and depression for 3 years. They found an increasing trend in BMI (28) which may reflect the chronic effect of anxiety and depression. Burke and Storch in a meta-analysis of weight status in children and adolescents with anxiety revealed a small relationship (r = 0.8; p < 0.001), and identified age and gender as the main moderators in this association (29). Also, they concluded that type of study and methodological variations may influence the results (29).

Tic disorder and enuresis were mostly observed in obese and overweight boys in this study. Degrauw et al. pointed out the possible effects of neuroleptic medications in short-term increasing body weight in children and adolescents with Tourette syndrome. They reported 13.5 kg weight gain as a result of low doses of neuroleptic drugs such as risperidone or pimozide (30). Consumption of medicines in severe cases is plausible, but, unfortunately, we did not gather the medication regiments in the study population. 

On the other hand, generalized anxiety is mostly observed in underweight girls, while ODD, depression, and mental retardation are mostly observed in obese girls. Vila et al revealed that about 16% of children and adolescents with obesity experience disruptive behavior disorders (DBD) (24). Like boys, girls with at least one psychiatric disorder were mostly overweight. 

The highest numerical BMI rates are observed among children and adolescents with alcohol abuse disorder, mania, and panic disorder, respectively. When we subgrouped the figures by gender, the first rank of the highest BMI in girls with alcohol abuse remained but it fell to the second rank in boys (figures 2 and 3). Vagstrand et al showed that the relationship of alcohol consumption and increasing body weight is gender-dependent and mostly observed among girls (31). McCarty et al have reported that depression increases the chance of alcohol use, especially in women, increasing the probability of obesity in these patients (18). Fuglestad et al investigated the rate of obesity and overweight among children and adolescents with fetal alcohol spectrum disorders in a case control design. They concluded that the fetal alcohol spectrum disorders may increase the rates of overweight and obesity (32). Alcohol with seven kcal/g energy increases the total calorie intake of consumers. Furthermore, metabolic and endocrine changes may be responsible for obesity in these children. Rojdmark et al revealed that alcohol consumption may decrease leptin (33). Koob bane et al (34) and Yeomans and Gray (35) revealed that GABAergic, opioid, and serotonergic mechanism increase appetite in those with alcohol use disorder. Mania had the second rank and hypomania the fifth rank in the status of BMI among common psychiatric disorders in children and adolescents. When we subgrouped the graphs by gender, the rank of hypomania increased to third in girls. In boys, the rank of mania has fallen to third and hypomania to sixth, but it still remained among high BMI rates. Mostafavi et al reported higher body mass index in female participants with bipolar disorder taking antipsychotic medications (36). Fleet‐Michaliszyn et al revealed that female participants with bipolar mood disorder tend to deposit more abdominal fat compared with their healthy peers (37). Goldstein et al studied the association of bipolar disorder and BMI. They showed that prevalence of obesity is higher among those with bipolar disorders. Furthermore, they revealed that this association is positively moderated by female gender (38). Reilly-Harrington et al also pointed out the obesity issue in patients with bipolar mood disorder. Binge eating and lack of control over dietary intake may be responsible for increasing calorie intake and body weight /BMI in these patients. Furthermore, Reilly-Harrington et al pointed out the increasing weight issue of common medications prescribed for treatment of bipolar mood disorders (39). 

Children and adolescents with panic disorder were among the top three highest mean BMI in this study. When BMIs were categorized according to gender, the rank increased to the first position in boys (figure 2). Lykouras et al reported that obesity is positively associated with panic disorders. They also reported a positive association of social phobia, specific phobia, and generalized anxiety disorder with obesity, especially among female participants (40). Also, in a nationally representative sample, Pagoto et al have shown a positive correlation between posttraumatic stress disorder and increased BMI, but this association was not necessarily mediated by binge eating (41). 

The lowest BMI rates were found among children and adolescents with ADHD, separation anxiety disorder, and enuresis. When the participants were categorized by gender, the lowest BMIs in girls were related to ADHD, encopresis, and separation anxiety disorder. In boys, the lowest BMIs were related to separation anxiety disorder and ADHD. 

The studies of association of ADHD and BMI in children and adolescents are conflicting. Some studies have reported higher BMI and obesity (42). Unregulated eating behaviors and common neurobiological disturbances, eg, dopaminergic pathway has been observed in obesity and ADHD, which may be responsible for this association (42). Some studies have reported lower BMI in ADHD children, which mostly indicate that the role of medication is an important confounding factor in this association. Stimulant medications that are usually consumed by ADHD children may alter the dietary intake and body weight in these children (43, 44). Díez-Suárez et al. have also reported slight weight loss followed by administration of methylphenidate in children with ADHD (45). Unfortunately, we did not gather the information on possible medication intake in this study. 

The number of investigations on the association of separation anxiety disorder and BMI in children and adolescents is surprisingly very low. Thus, further research is needed in this filed to clarify the kind of association and its mechanism. However, poor child care and lower dietary intake may be responsible for lower BMI that we observed in boys and girls with separation anxiety disorder.

Most previous studies have focused on adult population and ignored children and adolescents. However, in the present study, the participants were children and adolescents aged 6 to 18 years. The participants of this survey were from rural and urban areas and evenly selected from both genders and three age groups. Hence, the findings have the potential to be generalized to the community of children and adolescents. 

BMI: Body Mass Index, Depression D: depression disorder, Panic D: panic disorder, SAD: separation anxiety disorder, So-Ph: social phobia, Sp-Ph: specific phobias, Ag-Ph: agoraphobia, GAD: generalized anxiety disorder, OCD: obsessive compulsive disorder, PTSD: post-traumatic stress disorder, ADHD: attention deficit hyperactivity disorder, ODD: oppositional defiant disorder, CD: conduct disorder, Tic D: tic disorder, MR: mental retardation.

BMI: Body Mass Index, Depression D: depression disorder, Panic D: panic disorder, SAD: separation anxiety disorder, So-Ph: social phobia, Sp-Ph: specific phobias, Ag-Ph: agoraphobia, GAD: generalized anxiety disorder, OCD: obsessive compulsive disorder, PTSD: post-traumatic stress disorder, ADHD: attention deficit hyperactivity disorder, ODD: oppositional defiant disorder, CD: conduct disorder, Tic D: tic disorder, MR: mental retardation.

## Limitation

This study only provided a snapshot of BMI status in different psychiatric disorders, so longitudinal studies are needed to infer causality. One of the limitations in this study was the possible observer bias; however, its effect was decreased by accurate data screening and cleaning processes. Moreover, our study was limited by lack of information about drug history and lack of history about other physical disorders. 

## Conclusion

This was the largest study of its kind on BMI in children and adolescents with psychiatric disorders that provided an overall picture and snapshot of BMI status in different psychiatric disorders according to gender. Furthermore, the findings of this study are clinically important and in a multidisciplinary approach, they draw the attention of child psychiatrists to the status of BMI in their clients.
